# Association between Ureteral Clamping Time and Acute Kidney Injury during Robot-Assisted Radical Cystectomy

**DOI:** 10.3390/curroncol28060418

**Published:** 2021-11-29

**Authors:** Yudai Ishiyama, Tsunenori Kondo, Hiroki Ishihara, Kazuhiko Yoshida, Junpei Iizuka, Kazunari Tanabe, Toshio Takagi

**Affiliations:** 1 Department of Urology, Tokyo Women’s Medical University Medical Center East, 2-1-10 Nishiogu, Arakawa-ku, Tokyo 1168567, Japan; ishiyama.yudai@twmu.ac.jp (Y.I.); ishihara.hiroki@twmu.ac.jp (H.I.); 2 Department of Urology, Tokyo Women’s Medical University, 8-1 Kawada-cho, Shinjuku-ku, Tokyo 1620054, Japan; yoshida.kazuhiko@twmu.ac.jp (K.Y.); jiizuka@twmu.ac.jp (J.I.); tanabe.kazunari@twmu.ac.jp (K.T.); takagi.toshio@twmu.ac.jp (T.T.)

**Keywords:** acute kidney injury, robotic surgery, bladder cancer, radical cystectomy

## Abstract

Robot-assisted radical cystectomy (RARC) is replacing open radical cystectomy (ORC) and requires clamping of the ureters, resulting in a predisposition to postrenal acute kidney injury (AKI). We investigated the association between ureteral clamping or its duration and acute/chronic postoperative kidney function. Patients who underwent radical cystectomy (robotic or open) at two tertiary institutions during 2002–2021 were retrospectively enrolled. In those who underwent RARC, the maximum postoperative percentage serum creatinine level (%sCre) change was plotted against ureteral clamping duration. They were divided into two groups using the median clamping time (210 min), and the maximum %sCre change and percentage estimated glomerular filtration rate (%eGFR) change at 3–6 months (chronic) were compared between the ORC (no clamp), RARC < 210, and RARC ≥ 210 groups. In 44 RARC patients, a weak correlation was observed between the duration of ureteral clamping and %Cre change (R^2^ = 0.22, *p* = 0.001). Baseline serum creatinine levels were comparable between the groups. However, %sCre change was significantly larger in the RARC ≥ 210 group (N = 17, +32.1%) than those in the RARC < 210 (N = 27, +6.1%) and ORC (N = 76, +9.5%) groups (both, *p* < 0.001). Chronic %eGFR change was comparable between the groups. Longer clamping of the ureter during RARC may precipitate AKI; therefore, the clamping duration should be minimized.

## 1. Introduction

Radical cystectomy (RC) remains the gold standard treatment for patients with muscle-invasive or high-risk non-muscle-invasive urinary carcinoma of the bladder and is recommended in various guidelines globally [1,2]. While open RC (ORC) is still relevant, minimally invasive surgery, such as robot-assisted RC (RARC), results in less blood loss and better postoperative recovery [3].

One of the distinct features of minimally invasive surgery in comparison with the open approach in RC is the clamping of ureters during the intracorporeal procedures. Laboratory studies have reported that ureteral obstruction increases the intratubular pressure and may worsen renal function [4]. In the phase 3 RAZOR trial, the rate of acute renal failure (as defined in that study) was 11%, and no difference in its incidence was observed in comparison with ORC [3]. Additionally, mid-term renal function is reported to be comparable between ORC and RARC or across different urinary diversions [5]. However, the level of evidence is not very strong, and therefore, maximum effort is warranted to avoid acute kidney injury (AKI) [5]. Both AKI and long-term renal function outcomes are attributed to various factors, including preoperative renal function, hypertension, and diabetes; therefore, it is quite challenging to evaluate the effects of ureteral clamping on short-term renal function.

In this study, we aimed to assess the effects of the duration of ureteral clamping time (UCL) during RARC on the postoperative renal function with a focus on the acute phase (AKI) and the chronic phase.

## 2. Materials and Methods

### 2.1. Patient Selection

We retrospectively recruited 199 patients who underwent RC at two tertiary institutions, which included those who underwent RARC between April 2018 and July 2021 (N = 83) and those who underwent ORC between May 2004 and July 2021 (N = 116). After excluding patients who underwent simultaneous nephroureterectomy (N = 32), those with end-stage renal failure (N = 8), those without complete operative video recording during RARC (N = 32, as UCL could not be measured), and those with insufficient clinical or follow-up data (N = 7), 120 (44 RARC, 76 ORC) patients were finally analyzed. All clinical and laboratory data were obtained from the institutions’ electronic databases and patient medical records.

### 2.2. Study Design

First, in patients who underwent RARC, the duration of UCL was plotted against the maximum postoperative percentage change in serum creatinine (%sCre change) to assess acute kidney failure. UCL was also evaluated in relation to the maximum postoperative percentage change in estimated glomerular filtration rate (%eGFR change) at 3–6 months to assess chronic renal function. Next, these RARC patients were classified into groups as is stated in Section 2.5. Statistical Analysis. The following outcomes were assessed between the RARC groups of patients, as well as between the two RARC and ORC groups: postoperative maximum sCre; %sCre change; AKI according to Kidney Disease: Improving Global Outcomes (KDIGO) guidelines (serum creatinine compared with preoperative reference: stage 1: 1.5–2.0 times; stage 2: 2.0–2.9 times; and stage 3: ≥3.0 times [6]); eGFR at 3–6 months postoperatively; %eGFR change at 3–6 months; operative time (OT); estimated blood loss (EBL, mL). We utilized sCre to evaluate AKI and eGFR for chronic renal function change as is suggested in the international guidelines [6,7].

Finally, the relationship between possible factors and %sCre change or %eGFR change at 3–6 months were each analyzed.

### 2.3. Surgery and Perioperative Care

Both RARC and ORC were performed as described previously [8,9,10,11,12]. Since 2018 (Japanese national insurance system coverage), RARC has been the preferred surgery except for patients with contraindications, such as those with a history of multiple abdominal operations. The type of urinary diversion, including intracorporeal urinary diversion (ICUD) or extracorporeal urinary diversion (ECUD) during RARC, was determined by the operating surgical team based on the patient’s preoperative status, including comorbidities, surgical history, and preference. In both RARC and ORC, cystectomy was performed first, followed by lymph node dissection and urinary diversion. In RARC, the ureter was clamped halfway through cystectomy and unclamped during urinary diversion after pelvic lymphadenectomy, which was performed with an extended template in a majority of cases [13]. UCL was measured retrospectively using the recorded operation video and defined as the duration between the last clamping of either of the ureters and the unclamping of the first ureter. In ORC, the ureter was catheterized using a 6-Fr single-J catheter immediately after ligation. Therefore, there was no UCL. The number of surgeons involved included 10 in ORC and six in RARC.

Postoperative management was generally performed according to the Enhanced Recovery After Surgery (ERAS) protocol [14]. Briefly, fluid intake was started on the day of the surgery, and oral nutrition was initiated as soon as it could be tolerated by patients in both RARC and ORC groups. Parental nutrition was initiated at the physicians’ discretion if oral nutrition could not be initiated within 5–7 days.

### 2.4. Neoadjuvant and Adjuvant Chemotherapy

Neoadjuvant chemotherapy was considered when the patients were fit and when lymph node or surrounding tissue involvement was suspected in imaging studies. Adjuvant chemotherapy was offered when lymph node metastasis or local invasion was confirmed in the surgical specimen. All final decisions were made by the surgical team after considering the patients’ comorbidities and preferences. While several chemotherapy regimens were used, all were based on either cisplatin or carboplatin.

### 2.5. Statistical Analysis

Continuous variables were analyzed using the Mann–Whitney U-test and expressed as medians and interquartile range (IQR). β-coefficient was calculated using linear regression models. Categorical variables were analyzed using the χ^2^ or Fisher’s exact tests with odds ratio (OR) and 95% confidence interval. The median UCL value was used as the cutoff to classify the RARC patients into two groups. The patients were also classified into three groups according to UCL quartiles (shortest quartile, longest quartile, and between). Assessment of the relationship between possible factors and %sCre change or %eGFR change at 3–6 months were first performed using univariate analysis using the following variables: age, baseline eGFR, presence of hydronephrosis at the time of surgery, hypertension (on medications), diabetes, OT, EBL, perioperative urinary tract infections (for %eGFR change only), and UCL. Multivariate analyses were also performed in cases in which ≥ two significant factors were identified. Multivariate linear regression models were used to adjust the β-coefficient in cases when two or more significant factors were identified univariate analysis. All analyses were performed using JMP v14.0 (SAS Institute, Cary, NC, USA). Differences were considered statistically significant at *p*-values < 0.05.

## 3. Results

### 3.1. Patient Characteristics

The patients’ characteristics are summarized in Table 1. The median age at surgery in the RARC group was 74.3 years (IQR, 69.0–80.0), and 77.3% were male. All preoperative parameters were equally distributed between both sexes, except for the presence of hydronephrosis (Male: 26.1%, Female: 46.9%, *p* = 0.034). The median preoperative eGFR was 52.0 (45.1–70.1) mL/min/1.73 m^2^. The prevalence of hypertension and diabetes were 27.3% and 29.5%, respectively. Hydronephrosis was observed in 36.4% of patients. The type of urinary diversion used included ileal conduit (79.5%), orthotopic neobladder (4.5%), and ureterocutaneostomy (15.9%); additionally, 28 (63.6%) procedures used ICUD, and 18 (40.9%) procedures used ECUD.

Patients who underwent RARC were significantly older (74.3 (69.0–80.0) years) than those who underwent ORC (69.2 (58.5–75.6) years, *p* = 0.001) and had marginally lower eGFR (52.0 (45.1–70.1) vs. 62.5 (47.6–81.2) mL/min/1.73 m^2^, respectively, *p* = 0.065). There were also significant differences in the American Society of Anesthesiologists (ASA) score (*p* = 0.043) and the type of urinary diversion (*p* < 0.001); more patients were treated with a neobladder in the ORC group than those in the RARC group. All other baseline characteristics were comparable between the RARC and ORC groups of patients.

### 3.2. Association between UCL and Acute/Chronic Phase Renal Function Change in RARC

**Figure 1:**
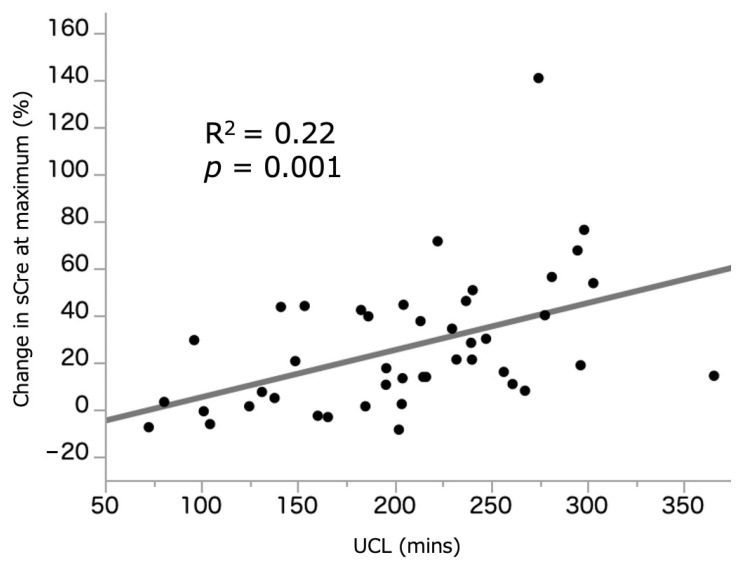
Correlation between UCL and %sCre change at maximum. sCre: serum creatinine. UCL: ureteral clamping time.

**Figure 2:**
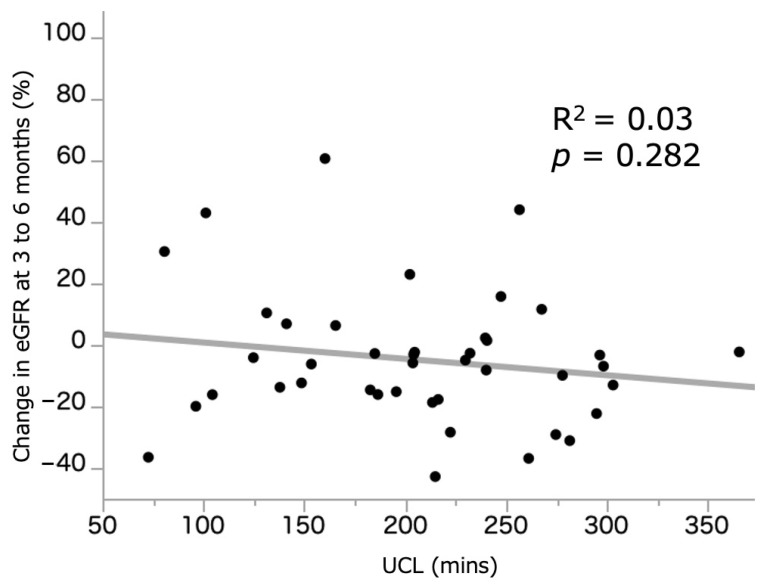
Correlation between UCL and %eGFR change at 3 to 6 months. eGFR: estimated glomerular filtration rate. UCL: ureteral clamping time.

Figure 1 illustrates the relationship between UCL (209.1 (155.2–254.4) min) and %sCre change. A weak but significant correlation was observed between the two parameters (R^2^ = 0.22, *p* = 0.001). Figure 2 illustrates the relationship between UCL and %eGFR change at 3–6 months, which demonstrated no significant correlation (R^2^ = 0.03, *p* = 0.282). In those who underwent ileal conduit, there was no difference in the UCL between the intracorporeal (*n* = 19, 229.9 (195.8–256.7) min) and extracorporeal (203.1 (166.5–255.9) min) procedures (*p* = 0.227). The median and average UCL was 209.1 (155.2–254.4) and 206.6 (±66.7) min, respectively. We, therefore, set the median cutoff value of UCL at 210 min, and patients who underwent RARC were divided into the RARC < 210 and RARC ≥ 210 groups. Additionally, 155 and 245 min were used as a cutoff value of shortest and longest quartiles.

**Figure 3:**
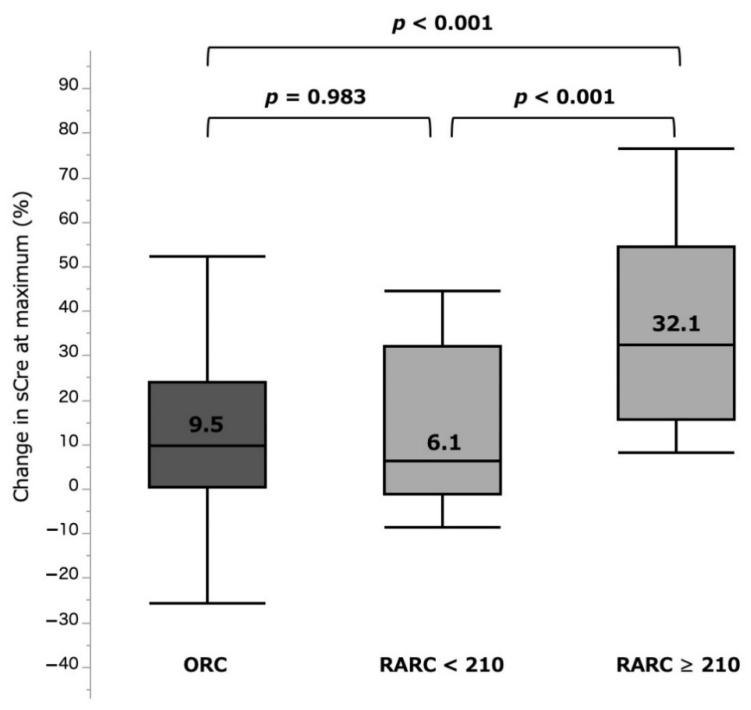
%sCre change at maximum by three surgical types. ORC: open radical cystectomy. RARC: robot-assisted radical cystectomy. sCre: serum creatinine.

**Figure 4:**
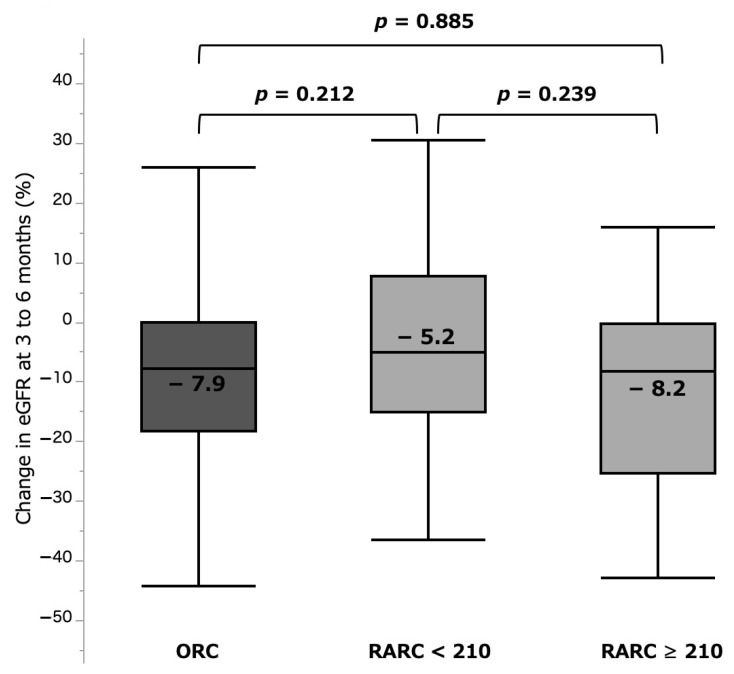
%eGFR change at 3 to 6 months by three surgical types. eGFR: estimated glomerular filtration rate. ORC: open radical cystectomy. RARC: robot-assisted radical cystectomy.

The %sCre change was significantly larger in the RARC ≥ 210 group (32.1 (15.5–54.3)%) when compared with those in the RARC < 210 group (6.1 (−1.3 to 32.0)%, *p* < 0.001) and ORC group (9.5 (0.2–23.9)%, *p* < 0.001). There was no significant difference between the RARC < 210 and ORC groups (*p* = 0.983) (Figure 3, Table 2). Incidence of AKI (any grade) was also significantly higher in the RARC ≥ 210 group (31.8%), compared with those in the RARC < 210 (0.0%, *p* < 0.001) and ORC (2.6%, *p* < 0.001) groups. In contrast, %eGFR change at 3–6 months was −8.2 (−25.4 to −0.4)%, −5.0 (−15.2 to 7.7)%, and −7.9 (−18.3 to −0.2)% in the RARC ≥ 210, RARC < 210, and ORC groups, respectively, with no significant differences between them (Figure 4, Table 2).

**Figure 5:**
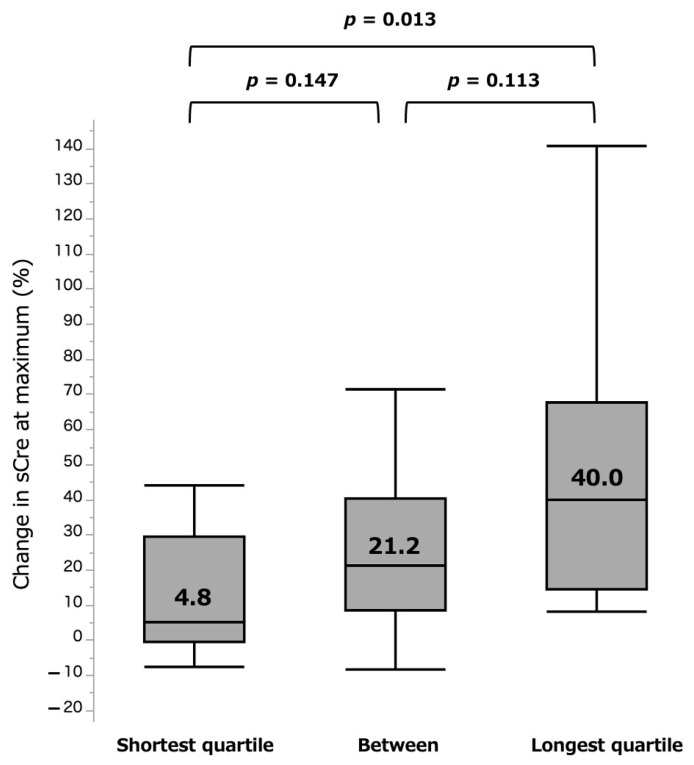
%sCre change at maximum by three surgical types. sCre: serum creatinine.

**Figure 6:**
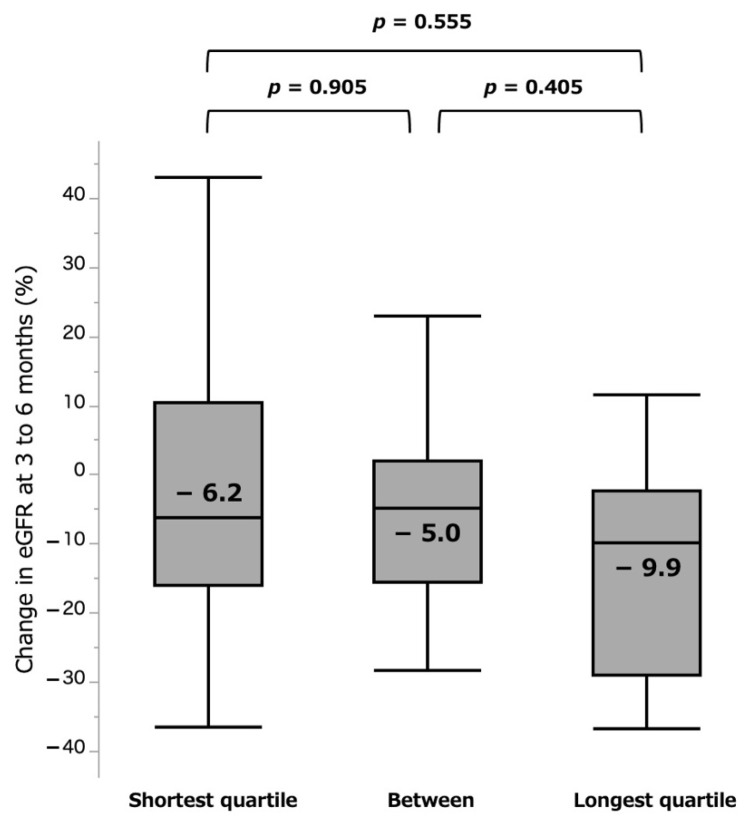
%eGFR change at 3 to 6 months by quartile groups. eGFR: estimated glomerular filtration rate.

In comparative analyses of the groups of quartiles, there were further distinct differences of 4.8% vs. 40.0% for shortest and longest quartile groups (*p* = 0.013) at the acute phase (Figure 5). For the chronic phase, the shortest group (−6.2%) showed less eGFR loss than the longest (−9.9%) quartile group, but the difference was again not significant (*p* = 0.555, Figure 6).

### 3.3. Relationship between UCL and Other Outcomes

Surgical time was significantly shorter in the RARC < 210 group (392.0 (278.8–452.8) min), compared with that in the RARC > 210 (466.0 (430.8–536.5) min, *p* < 0.001) and ORC (463.0 (356.0–548.0) min, *p* = 0.003) groups. There was no significant difference between the RARC ≥ 210 and ORC groups (*p* = 0.395). EBL was the lowest in the RARC < 210 (103.5 (52.3–262.5) mL) group in comparison with the RARC ≥ 210 (275.0 (160.3–470.0) mL, *p* < 0.001) and ORC (1080.0 (527.5–2417.5) mL, *p* < 0.001) groups. The difference between the RARC ≥ 210 and ORC groups was also statistically significant (*p* < 0.001) (Table 2).

### 3.4. Factors Affecting the Acute/Chronic Phase Renal Function Change in RARC

We analyzed the variables that may be related to acute %sCre change and chronic %eGFR change. In univariate analysis of acute %sCre change, UCL (continuous value, β-coefficient 0.20 (standard error (SE) 0.06), *p* = 0.001) age (continuous value, β-coefficient 0.59 (SE 0.34), *p* = 0.061), and surgical time (continuous value, β-coefficient 0.09 (SE 0.04), *p* = 0.025) were identified as significant factors. In multivariate analysis, UCL was demonstrated to be independently associated (β-coefficient 0.25, (SE 0.11), *p* = 0.023) (Table 3). In univariate analysis of chronic %eGFR change, only preoperative baseline eGFR was identified as an associated factor (β-coefficient −0.43 (SE 0.18), *p* = 0.019) (Table 4).

## 4. Discussion

In this study, a significant association between UCL and %sCre change was observed in the AKI phase. When stratified into two groups based on the median UCL time (210 min), patients with UCL ≥ 210 min had a greater increase in %sCre and were more likely to develop AKI, compared with those with UCL < 210 min or who underwent ORC. No association between UCL and chronic (3–6 months) renal function was evident.

Acute kidney damage caused by urinary tract obstruction is recognized as postrenal AKI. There are several proposed mechanisms for this phenomenon, including increased intratubular pressure resulting in a decline in GFR [4], and that obstruction may lead to impairment of renal circulation and inflammation [15], both of which contribute directly to GFR loss [16]. These reactions later result in fibrotic changes in the renal parenchyma [17], thus precipitating the chronic loss of renal function. Animal laboratory model data suggest a relationship between the duration of ureteral obstruction and the severity of the damage. In rats, renal function evaluated using 99mTc-dimercaptosuccinic acid (DMSA) uptake decreased from 35% at baseline (before ureter ligation) to 13% after 24 h and further to 1.5% after 31 days. If the ureter was freed after 10 days, DMSA uptake recovered from 7% to 15%, whereas the recovery was only from 1.5% to 2% when ligation was prolonged to 30 days [18].

However, in humans, the exact relationship between kidney damage and ureteral obstruction, bilateral obstruction particularly, has been poorly investigated largely due to the difficulty in collecting patient data under such conditions. Furthermore, whether very limited UCL, as short as 100–300 min (as described in our study), affects acute renal function was unknown. The current finding that UCL is correlated with acute kidney damage is a novel addition to the existing knowledge. We set the UCL cutoff value at 210 min to identify significant differences in renal function and AKI incidence. There is no rationale yet to support this cutoff value; therefore, further studies are warranted to identify the optimal UCL threshold. Furthermore, the finding that eGFR recovery at 3–6 months was comparable between the groups irrespective of UCL is partly in line with previous laboratory data on return of renal function following ureteral obstruction of 4–14 days [19,20].

There was one patient in the RARC group who had a nephrostomy placed prior to RC and left open during the operation. UCL duration for this patient was 278 min (i.e., >210), and he experienced a rather large increase in sCre (40.0%). Theoretically, the risk of AKI would be considerably low if one kidney was freed from postrenal occlusion [21], but this specific finding does not necessarily support this supposition. We believe extensive research is required to answer if the preoperative placement of nephrostomy can protect patients from AKI.

There is a growing body of evidence to support AKI prevention for multiple purposes. Strong interconnection has been reported between AKI and chronic kidney disease (CKD) [22]. Additionally, AKI has been reported to have independent effects on perioperative complications and mortality [23]. In terms of CKD progression, we could not detect any differences in renal function at 3–6 months postoperatively between the groups. This finding is distinct from those of studies that have investigated the association between AKI and CKD after partial nephrectomy, in which AKI is possibly caused by loss of nephrons and ischemia [24]. The differences in the pathophysiology may explain this discrepancy, while the small sample size and short follow-up period could have also contributed to it.

Apart from ureteral obstruction, RC is a procedure with an inherent considerable risk of damage to renal function. Furrer et al. reported that 11% of patients in their ORC cohort experienced AKI [25]. This number is comparable to the one observed in the current study. Lone et al. reported that 64% of patients who underwent either ORC, RARC with ICUD, or RARC with ECUD experienced a decline in eGFR ≥ 10 mL/min/1.73 m^2^ with no differences between the surgery type [5]. It should be borne in mind that UCL in RC has not been extensively studied since it was only after the utilization of laparoscopic and robotic technologies that surgeons began ureteral clamping to continue intracorporeal procedures after ligating the ureters [9].

Data have revealed possible gender discrepancies in bladder cancer management and outcomes. In a recent review, females were shown for significantly longer postoperative stay, operative time, more blood loss, and a higher rate of mortality or complications [26]. In the present study, the preoperative parameters were comparable between males and females. Furthermore, %sCre change at acute phase was larger for males (14.0%) compared with females (9.7%) with no significance (*p* = 0.141), while occurrence of AKI were more frequently observed among males (10.2% vs. 0.0%, *p* = 0.016). %eGFR change at chronic phase was comparable between sexes (non-significant). As a result, we could not detect any signs that females are more prone to AKI after RC. We believe a further focus on these disparities is needed. 

After confirming that AKI is likely to occur in those with prolonged UCL, we should emphasize that preventing AKI after RC is of paramount importance due to several reasons. First, AKI in itself is a significant risk factor for both short-term and long-term cardiovascular events [27]. Second, patients with bladder cancer are generally older (over 70 years) at the time of diagnosis [28] and may be prone to CKD following AKI [29,30]. In fact, the median age of our patients was ≥70 years. Third, renal function impairment may limit the therapeutic options during the perioperative period, thus possibly resulting in serious adverse events. Therefore, UCL time should be minimized to reduce the risk of postoperative AKI in RARC. The time required to deliver safe and effective RARC (as well as ICUD) requires a certain learning curve [31,32]. In cases of planned lymphadenectomy, performing lymphadenectomy before cystectomy may serve as a possible strategy to shorten UCL time. As all patients in this study underwent lymphadenectomy after cystectomy, further investigations are required to justify the optimal sequence. At the least, ureteral clamping should be withheld until the bladder is mobilized as much as possible. For procedures of urinary diversion (ICUD or ECUD) used in the ileal conduit, we did not observe any difference in the UCL time between the techniques. This finding may show that trained surgeons with adequate training can perform ICUD with UCL as efficiently as the traditional ECUD.

Our study has some limitations. This was a retrospective cohort study involving a relatively small number of patients, which may have introduced selection bias. Second, the effects of urinary diversion on UCL time or renal function could not be evaluated due to the small number of patients in the RARC cohort. Third, interpretation of our analysis between the RARC and ORC cohort ought to be carried out with caution, as their clinical background differed in several aspects. Further studies, ideally prospective and with longer follow-up periods, are warranted to corroborate our findings.

## 5. Conclusions

This retrospective analysis showed that UCL time during RARC was significantly associated with acute renal function loss and AKI. Although its effects on chronic renal function were limited, effort should be incorporated to minimize the UCL time and protect patients from potential AKI and its sequelae.

## Figures and Tables

**Table 1:** Patient characteristics.

	All (*n* = 120)	RARC (*n* = 44)	ORC (*n* = 76)	*p*-Value
Age at surgery, median (IQR)	71.6 (62.4–77.5)	74.3 (69.0–80.0)	69.2 (58.5–75.6)	0.001
Sex, male (%)	88 (73.3)	34 (77.3)	54 (71.1)	0.454
ASA score				0.043
1	12 (10.0)	1 (2.3)	11 (14.5)	
2	86 (71.7)	37 (84.1)	49 (64.5)	
3	21 (17.5)	6 (13.6)	15 (19.7)	
4	1 (0.8)	0 (0.0)	1 (1.3)	
Diabetes mellitus	27 (22.5)	13 (29.5)	14 (18.4)	0.164
Hypertension	34 (28.3)	12 (27.3)	22 (28.9)	0.844
BMI, median (IQR)	22.2 (19.8–24.6)	22.4 (19.8–24.8)	21.9 (19.8–24.6)	0.226
Preoperative sCre, median (IQR)	0.9 (0.8–1.2)	0.9 (0.8–1.2)	0.9 (0.7–1.2)	0.174
Preoperative eGFR, median (IQR)	59.5 (45.6–76.7)	52.0 (45.1–70.1)	62.5 (47.6–81.2)	0.065
Neoadjuvant chemotherapy	60 (50.0)	21 (47.7)	39 (51.3)	0.705
Preoperative T stage				0.278
<T1	32 (26.7)	9 (20.5)	23 (30.3)	
T2	49 (40.8)	20 (45.5)	19 (25.0)	
T3	25 (20.8)	12 (27.3)	13 (17.1)	
Preoperative hydronephrosis	38 (31.7)	16 (36.4)	22 (28.9)	0.402
Type of urinary diversion				<0.001
Ileal conduit	74 (61.7)	35 (79.5)	39 (51.3)	
Neobladder	29 (24.2)	2 (4.5)	27 (35.5)	
Ureterocutaneostomy	17 (14.2)	7 (15.9)	10 (13.2)	
ICUD or ECUD				
ICUD	-	28 (63.6)	-	-
ECUD	-	18 (40.9)	-	-

ASA: American Society of Anesthesiologists; BMI: body mass index; ECUD: extracorporeal urinary diversion; eGFR: estimated glomerular filtration rate; ICUD: intracorporeal urinary diversion; IQR: interquartile range; ORC: open radical cystectomy; RARC: robot-assisted radical cystectomy; sCre: serum creatinine.

**Table 2:** Surgical outcomes.

	ALL (*n* = 120)	RARC < 210 (*n* = 22)	RARC ≥ 210 (*n* = 22)	ORC (*n* = 76)	*p*-Value RARC < 210 vs. RARC ≥ 210	*p*-Value RARC < 210 vs. ORC	*p*-Value RARC ≥ 210 vs. ORC
Postop maximum sCre	1.1 (0.8–1.4)	1.0 (0.8–1.3)	1.3 (1.2–1.5)	1.0 (0.8–1.3)	0.005	0.414	<0.001
Change in sCre at maximum (%)	13.8 (3.0–30.5)	6.1 (−1.3 to 32.0)	32.1 (15.5–54.3)	9.5 (0.2–23.9)	<0.001	0.983	<0.001
AKI (stage)					<0.001	0.310	<0.001
no AKI	111 (92.5)	22 (100.0)	15 (68.2)	74 (97.4)			
stage 1	8.0 (6.7)	0 (0.0)	6 (27.3)	2 (2.6)			
stage 2	1.0 (0.8)	0 (0.0)	1 (4.5)	0 (0.0)			
Postoperative eGFR at 3 to 6 months	52.4 (39.3–67.3)	56.0 (41.3–64.0)	44.5 (36.3–61.4)	53.1 (39.8–71.7)	0.148	0.997	0.147
Change in eGFR at 3 to 6 months (%)	−7.8 (−17.9 to 0.0)	−5.0 (−15.2 to 7.7)	−8.2 (−25.4 to −0.4)	−7.9 (−18.3 to −0.2)	0.239	0.212	0.885
Surgical time, median	454.0 (356.0–515.0)	392.0 (278.8–452.8)	466.0 (430.8–536.5)	463.0 (356.0–548.0)	<0.001	0.003	0.395
EBL, median	545.0 (217.5–1578.5)	103.5 (52.3–262.5)	275.0 (160.3–470.0)	1080.0 (527.5–2417.5)	<0.001	<0.001	<0.001

AKI: acute kidney injury; EBL: estimated blood loss; eGFR: estimated glomerular filtration rate; IQR: interquartile range; ORC: open radical cystectomy; RARC: robot-assisted radical cystectomy; sCre: serum creatinine.

**Table 3:** Univariate and multivariate linear regression for change in sCre at maximum (%).

	Univariate			Multivariate		
	β-Coefficient	SE	*p*-Value	β-Coefficient	SE	*p*-Value
Age (continuous)	−1.12	0.43	0.012	−0.70	0.46	0.137
Diabetes mellitus	−5.41	4.67	0.253			
Hypertension	1.64	4.85	0.736			
Preoperative eGFR (continuous)	0.17	0.24	0.494			
Surgical time	0.09	0.04	0.025	−0.07	0.07	0.292
EBL	0.01	0.01	0.614			
Hydronephrosis	2.15	4.48	0.634			
UCL (continuous)	0.20	0.06	0.001	0.25	0.11	0.023

EBL: estimated blood loss; eGFR: estimated glomerular filtration rate; sCre: serum creatinine; SE: standard error; UCL: ureteral clamping length.

**Table 4:** Linear regression for change in eGFR at 6 months (%).

	Univariate		
	β-Coefficient	SE	*p*-Value
Age (continuous)	0.59	0.34	0.061
Diabetes mellitus	5.87	3.48	0.100
Hypertension	−4.84	3.61	0.187
Preoperative eGFR (continuous)	−0.43	0.18	0.019
Surgical time	−0.02	0.03	0.483
EBL	0.01	0.01	0.501
Hydronephrosis	−3.52	3.38	0.303
Perioperative UTI	−2.80	3.35	0.411
UCL (continuous)	−0.05	0.05	0.282

EBL: estimated blood loss; eGFR: estimated glomerular filtration rate; SE: standard error; UCL: ureteral clamping length; UTI: urinary tract infections.

## Data Availability

The data presented in this study are available on request from the corresponding author. The data are not publicly available due to privacy restrictions.
